# Combining pretreatment plasma Epstein‐Barr virus DNA level and cervical node necrosis improves prognostic stratification in patients with nasopharyngeal carcinoma: A cohort study

**DOI:** 10.1002/cam4.2481

**Published:** 2019-09-12

**Authors:** Yu‐Yun Du, Dong‐Hua Luo, Xue‐Song Sun, Lin‐Quan Tang, Hai‐Qiang Mai, Qiu‐Yan Chen, Jing‐Hua Zhong, Dong‐Mei Mai, Wan‐Ru Zhang, Wen‐Hui Chen, Hao‐Yuan Mo

**Affiliations:** ^1^ Sun Yat‐sen University Cancer Center State Key Laboratory of Oncology in South China Collaborative Innovation Center for Cancer Medicine Guangzhou P. R. China; ^2^ Department of Nasopharyngeal Carcinoma Sun Yat‐sen University Cancer Center Guangzhou P. R. China; ^3^ Department of Oncology The First Affiliated Hospital of Gannan Medical University Ganzhou P. R. China; ^4^ Department of Oncology The First Affiliated Hospital, Jinan University Guangzhou China

**Keywords:** cervical node necrosis, cohort, EBV, nasopharyngeal carcinoma

## Abstract

This study aimed to evaluate the prognostic value of combining pretreatment Epstein‐Barr virus (EBV) DNA level and cervical node necrosis (CNN) for patients with nasopharyngeal carcinoma (NPC) receiving intensity‐modulated radiotherapy (IMRT). A total of 607 incident nonmetastatic NPC patients treated with IMRT ± chemotherapy were reviewed. Patients were divided into four groups based on EBV DNA level and CNN status. The primary endpoint was progression‐free survival (PFS). Kaplan‐Meier curves with log‐rank test were applied to compare survival outcomes and the Cox proportional model was used to identify independent prognostic factors. Pretreatment EBV DNA level and CNN status were independent prognostic factors. Patients in the low‐level EBV DNA group or non‐CNN group had significantly better 5‐year PFS. Multivariate analyses demonstrated that CNN was an independent prognostic factor for overall survival (OS) (HR = 1.927, 95% CI: 1.129‐3.290, *P* = .016), PFS (HR = 1.492, 95% CI: 1.005‐2.214, *P* = .047), distant metastasis‐free survival (DMFS) (HR = 1.661, 95% CI: 1.044‐2.644, *P* = .032), but not locoregional relapse‐free survival. EBV DNA levels correlated significantly with CNN with a correlation coefficient of .324 (*P* < .001). Compared with low‐level EBV DNA and non‐CNN grouping, high‐level EBV DNA and CNN grouping had poor PFS. The combined classification was an independent prognostic factor for OS (*P* < .001), PFS (*P* = .001), and DMFS (*P* = .018). Pretreatment plasma EBV DNA level and CNN status both closely correlated with prognosis of NPC patients in the IMRT era. Combined EBV DNA level and CNN status improves risk stratification and prognostic value.

## Graphical abstract

It is the first retrospective study that combined pretreatment plasma EBV DNA level and cervical node necrosis (CNN) status to assess the prognosis of nasopharyngeal carcinoma patients. We observed a significant association between plasma EBV DNA level and CNN status in the IMRT era. Compared with the low‐level EBV DNA and non‐CNN group, the high‐level EBV DNA and CNN group had poorer OS, progression‐free survival, and distant metastasis‐free survival. The Kaplan–Meier survival curves showed a significant difference in survival between the different risk groups according to the optimal cut‐off.

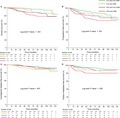

## 1 INTRODUCTION

Nasopharyngeal carcinoma (NPC), a group of malignant epithelial tumors with different etiopathogenesis and a broad range of clinical symptoms, are endemic in southern China and southeast Asia. On account of the concealed anatomical location of the nasopharynx, this cancer tends to be diagnosed at an advanced stage of the disease at a patient's first visit, and up to 85% of patients have regional lymph node metastasis at the time of diagnosis.^1^, ^2^ Regarding treatment, concurrent chemoradiotherapy with or without adjuvant chemotherapy is considered the standard modality. In addition to IMRT, survival outcome has greatly improved in NPC patients. However, approximately 20%‐30% of logically advanced NPC patients experience distant metastases.^3^, ^4^ If high‐risk patients can be selected before therapy and treated with intensive treatment, more person‐specific therapy can be provided.

Numerous efforts have been made to study tumor‐related prognostic factors for NPC patients in recent years. One of the most significant factors is pretreatment plasma Epstein‐Barr virus (EBV) DNA, which is clinically employed for the diagnosis, risk classification, dynamic monitoring, and prognosis of NPC.^5^, ^6^, ^7^, ^8^, ^9^ Pretreatment plasma EBV DNA level is an adverse independent prognostic factor for NPC patients.^7^ Chan et al demonstrated that one of the origins of EBV DNA was derived from tumor cell death.^10^, ^11^ Tumor cell necrosis may predict tumor hypoxia, and both of these factors, in addition to resistance to radiation are closely correlated with tumor volume.^12^, ^13^, ^14^, ^15^ As Ma et al demonstrated, total tumor volume, including primary tumor and regional nodes were significantly associated with pretreatment levels of EBV DNA in NPC.^12^ Lan et al also demonstrated that cervical node necrosis (CNN) is a negative prognostic factor for NPC.^16^ Based on these findings, we aimed to examine the association between EBV DNA level and CNN outcomes. High EBV DNA levels were hypothesized to be related to CNN and poor survival outcome. Consequently, pretreatment plasma EBV DNA levels correlate with CNN status and could become new prognostic tools in refining the current evaluation system. Here, we conducted a cohort study to assess whether a combination of plasma EBV DNA level and CNN status improved prognostic stratification in patients with NPC.

## 2 MATERIALS AND METHODS

### 2.1 Patient characteristics and pretreatment evaluation

A total of 607 incident, histologically confirmed, nonmetastatic NPC patients were enrolled in this study between December 2006 and December 2012. Patients were aged between 18 and 79 years (the median age was 44 years). Our retrospective study was approved by the ethics committee of Sun‐Yet Sen University, China. All patients underwent a pretreatment workup, which included complete medical history and hospital experience. Clinical examination include magnetic resonance imaging (MRI) of the nasopharynx and cervical regional, plasma EBV DNA assessment, pathologic biopsy or consultation, computed tomography (CT) or chest film, abdominal sonography, whole body bone scan or positron emission tomography‐computed tomography. These details were included in the cancer center hospital record. Furthermore, all patients had lymph node metastases, which included retropharyngeal lymph nodes or cervical nodes, without distant metastases.

The male to female (n = 439) ratio was 2.6:1. Patients had histologically confirmed, nonkeratinizing NPC, which included WHO type II (24 of 607; 4%) and III (583 of 607, 96%), respectively. These patients had no other serious illness or cancer‐associated disease, although 14.5% (88 of 607) had family history of NPC or other cancer type.

All patients were restaged according to the seventh edition of the Union for International Cancer Control/American Joint Committee on Cancer system.^17^ T‐stage, N‐stage, and other clinical characteristics of the patients are listed in Table 1.

**Table 1:** Patients characteristics (n = 607)

	EBV DNA ≤ 4000	EBV DNA ＞ 4000	*P*	Non‐CNN	CNN	*P*
Characteristics						
Total	386	221		424	183	
Gender			.707			.236
Male	277	162		313	126	
Female	109	59		111	57	
Age (years)			.023			.086
≤44	212	100		219	93	
＞44	174	121		205	90	
WHO pathology			.409			.256
Type II	14	10		14	11	
Type III	372	211		410	173	
T category*			.016			.574
T1	24	13		27	10	
T2	73	35		70	38	
T3	229	115		241	103	
T4	60	58		86	32	
N category*			.002			＜.000
N1	190	77		213	54	
N2	174	123		189	108	
N3	22	21		22	21	
Overall stage*			＜.000			.035
II	52	14		56	10	
III	259	130		268	121	
IV	75	77		100	52	
Type of therapy			.374			.565
RT	8	2		6	4	
CCRT	370	211		407	174	
NAC+RT	1	0		1	0	
NAC+CCRT	5	7		7	5	
CCRT+AC	2	1		3	0	

Abbreviations: AC, Adjuvant chemotherapy; CCRT, Concurrent chemoradiotherapy; CNN, cervical node necrosis; NAC, Neoadjuvant chemotherapy; non‐CNN, noncervical node necrosis; RT, Radiotherapy.

* The 7th AJCC/UICC staging system.

### 2.2 Treatment strategies

All patients were treated with 1.8‐2.27 Gy per fraction with five daily fractions per week using IMRT technique, for a total of 6‐7 weeks. Cumulative radiation doses were 60‐75 Gy to the gross tumor target of the nasopharynx (GTVnx) and 50‐70 Gy to the involved neck area (GTVnd). All potential regions of local target volume and cervical lymphatic nodes were treated with 50‐64 Gy or greater. To improve the therapeutic results in our NPC cohort, we adopted a standard treatment regimen, which included concurrent chemoradiotherapy (CCRT) ± neoadjuvant/adjuvant chemotherapy. Cisplatin‐based CCRT was the most common treatment method, which included cisplatin 80‐100 mg/m^2^ every 3 weeks for 2‐3 cycles.

### 2.3 Quantification of plasma EBV DNA level and assessment of CNN

**Figure 1:**
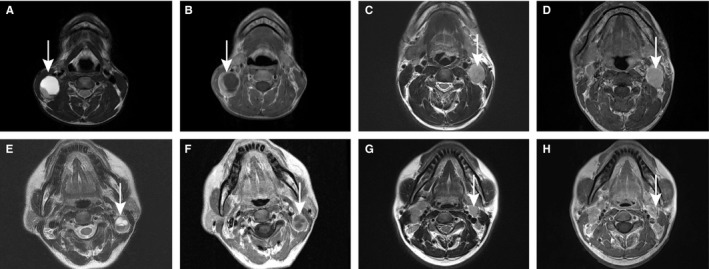
Lymph node status in four patient groups with nasopharyngeal carcinoma (NPC) by magnetic resonance images (MRI). (A) Axial T2‐weighted and (B) contrast‐enhanced T1‐weighted MRI in a 31‐y‐old man with pretreatment EBV DNA level of 6150 copies/mL and CNN; (C) Axial T2‐weighted and (D) contrast‐enhanced T1‐weighted MRI in a 42‐y‐old man with pretreatment EBV DNA level of 67 700 copies/mL and non‐CNN; (E) Axial T2‐weighted and (F) contrast‐enhanced T1‐weighted MRI in a 50‐y‐old woman with pretreatment EBV DNA level of 1460 copies/mL and CNN; (G) Axial T2‐weighted and (H) contrast‐enhanced T1‐weighted MRI in a 31‐y‐old man with pretreatment EBV DNA level of 1120 copies/mL and non‐CNN

Patient plasma EBV DNA concentrations were measured using a real‐time QPCR technique based on a proven system at the Department of Molecular Diagnosis, SYSUCC, as described in previous studies.^9^, ^18^ The cutoff value for pre‐EBV DNA was set at 4000 copies/mL, which was selected as the definition low and high EBV DNA levels, as has been previously established.^5^, ^19^, ^20^ All patients underwent MRI with a 1.5‐ or 3.0‐T system (Signa CV/i GE HealthCare) or CT with a dual‐helix CT Imager (Picker MX Marconi Twin flash). In our cancer center, two or three radiologists who specialized in head and neck cancer reviewed all MRI scans separately with no knowledge of patients’ clinical outcomes. The diagnostic criteria of CNN on MRI was based on T2‐weighted images showing a focal area of high‐signal intensity or T1‐weighted images displaying a focal low‐signal intensity with or without contrast‐enhanced images.^21^ In this study, we regarded the parapharyngeal lymph node as CNN (Figure 1). According to a previous study by Chung et al, the mean size of the greatest diameter of cervical lymph node, was 27 mm in patients with head and neck squamous cell carcinoma.^22^ In our study, we used 27 mm as the optimal cutoff value of the lymph node size. In addition, we performed a Spearman correlation analysis between CNN and the maximum diameter of the lymph node.

In this study, all patients were divided into four subgroups: high‐level EBV DNA and CNN grouping (HLE and CNN), high‐level EBV DNA and non‐CNN grouping (HLE and non‐CNN), low‐level EBV DNA and CNN grouping (LLE and CNN), and low‐level EBV DNA and non‐CNN grouping (LLE and non‐CNN).

### 2.4 Follow‐up

Follow‐up duration of our patients was calculated from the first day of therapy to the last day of death or examination. Patients were regularly checked once every 3 months in the first year, once every 3‐6 months, or annually during the subsequent years according to the results of the last checkup, or the time at which the patients were available for checkup especially if this had a bearing on outcome. The primary endpoint was progression‐free survival (PFS), and the secondary endpoints include overall survival (OS), locoregional relapse‐free survival (LRFS), and distant metastasis‐free survival (DMFS).

### 2.5 Statistical analysis

The Statistical Package for the Social Sciences, version 23.0(SPSS) was used for data analysis. The relationship between the plasma EBV DNA level and CNN was evaluated using the χ^2^ test. Kaplan‐Meier methods were used to analyze the OS, PFS, LRFS, and DMFS, while the log‐rank test was used to compare the differences between survival curves. Univariate and multivariate analysis employed the Cox proportional hazards model to determine significant prognostic factors, which included: gender, age, WHO histological type, smoking, NPC family history, T category, N category, and other relevant factors. Two‐tailed *P*‐values < .05 were considered statistically significant.

## 3 RESULTS

### 3.1 Patients characteristics and survival outcomes

Six hundred and seven patients were prospectively enrolled between December 2006 and December 2012, and patient characteristics are listed in Table 1. The median follow‐up duration was 88 months (range, 1.0‐137.0 months); 54 of 607 patients (8.9%) were lost to follow‐up. By the last follow‐up examination, 19.6% (n = 119) of patients experienced disease progress, 10.4% (n = 63) of patients died, while 7.1% and 12.7% developed local regional recurrence and distant metastases, respectively. Distant metastasis was the main reason for treatment failure. For all NPC patients, the 5‐year OS, PFS, LRFS, and DMFS rates were 92.6%, 85.0%, 95.4%, and 90.1%, respectively.

### 3.2 EBV DNA assessment and clinical outcome

**Figure 2:**
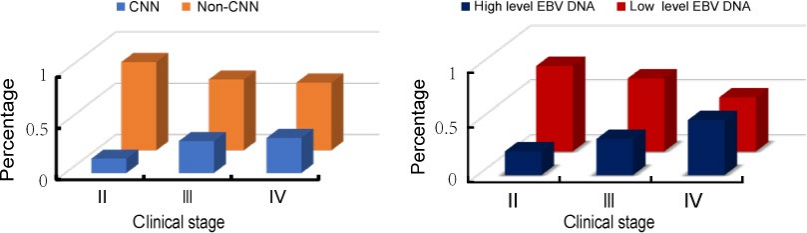
The percentage of CNN status(left) and EBV DNA level(right) in different clinical stages. *II, clinical stage II; III, clinical stage III; IV, clinical stage IV; *non‐CNN, non cervical node necrosis; CNN, cervical node necrosis

**Figure 3:**
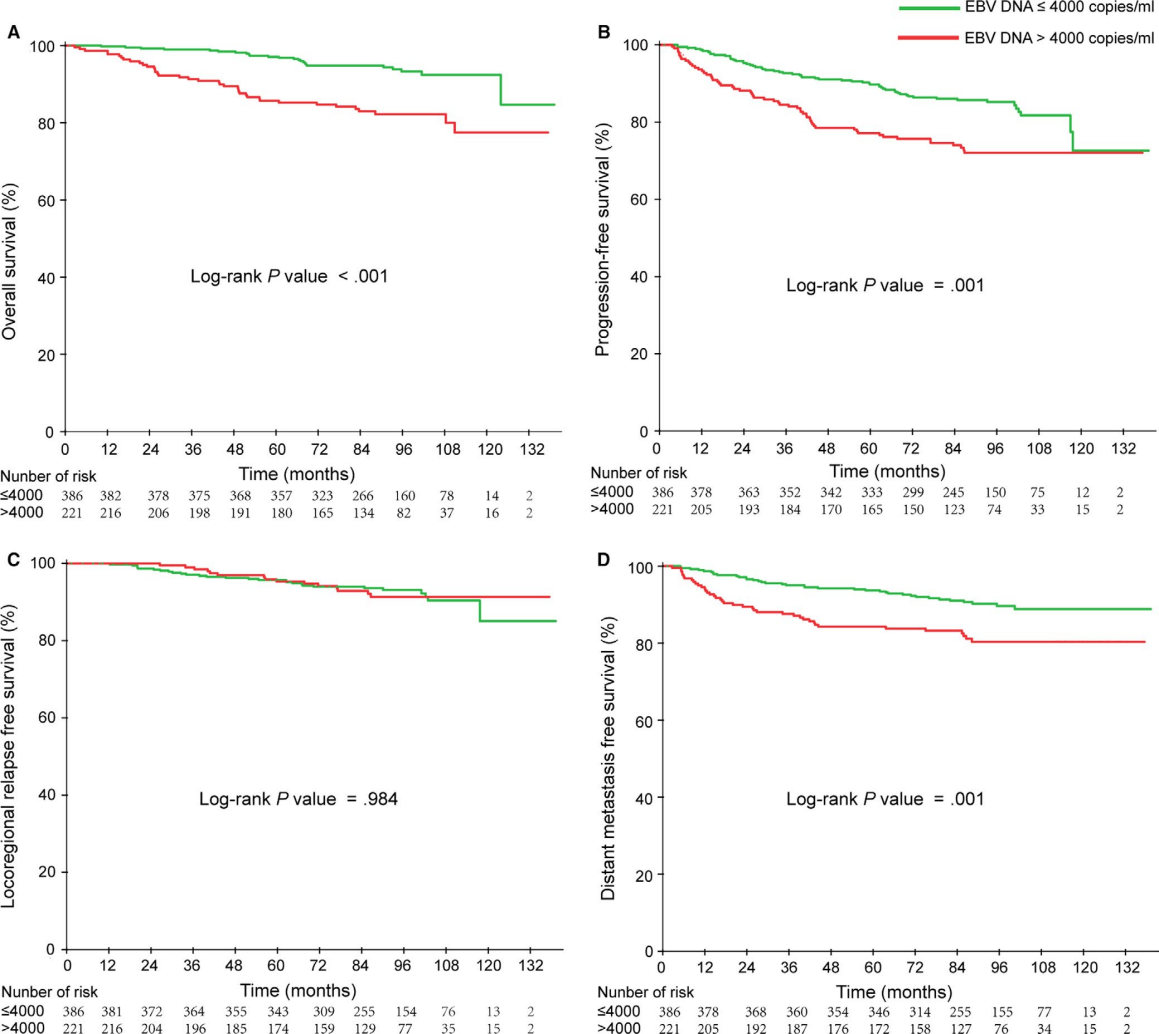
Kaplan‐Meier curves of overall (A), Progression‐free (B), Locoregional relapse‐free (C), and Distant metastasis‐free (D) survival outcomes for the 607 NPC patients stratified by EBV DNA level

Of the 607 eligible patients, we chose a cutoff level of 4000 copies/mL to define low and high EBV DNA levels. The percentage of patients in the low‐level EBV DNA group and high‐level EBV DNA group was 63.6% (386 of 607) and 36.4% (221 of 607), respectively. The incidence of low‐level EBV DNA and high‐level EBV DNA in clinical stages II, III, and IV was 78.1% and 21.9%, 66.8% and 33.2%, 49.3%, and 50.7%, respectively (Figure 2). Patients in the low‐level EBV DNA group had significantly better 5‐year OS (96.8% vs 85.3%, *P* < .000), PFS (89.6% vs 76.2%, *P* = .001), and DMFS (93.5% vs 83.9%, *P* = .001) than patients in the high‐level EBV DNA group. However, there was no difference in the 5‐year LRFS between the low‐level EBV DNA and high‐level EBV DNA groups (*P* = .986). Kaplan‐Meier survival curves for the four groups are shown in Figure 3.

In the multivariate analysis, the pretreatment high EBV DNA level was demonstrated to be an independent prognostic factor for OS (HR = 2.166, 95% CI: 1.258‐3.26, *P* = .005), PFS (HR = 1.619, 95% CI: 1.100‐2.382, *P* = .015), and DMFS (HR = 1.795, 95% CI: 1.107‐2.910, *P* = .018), but it was not statistically significant for LRFS.

### 3.3 Incidence of CNN and survival outcome

Among the entire cohort of 607 patients with positive lymph node metastases, the incidence of CNN was 183 (30.1%); age, gender, WHO pathology, T stage, N stage, overall stage, and type of treatment according to lymph node status is listed in Table 1. The incidence of CNN in N1, N2, and N3 stage was 20.2% (54 of 267), 36.4% (108 of 297), and 48.8% (21 of 43), respectively (*P* < .001). The rates of non‐CNN and CNN in clinical stages II, III, and IV were 85.9% and 14.1%, 68.8% and 31.2%, and 65.8% and 34.2%, respectively (Figure 2). There was no significant difference in the distribution of gender (*P* = .236), age (*P* = .860), WHO pathology (*P* = .256), T category (*P* = .574), or type of therapy (*P* = .565) while significant differences were observed for N category (*P* < .001) and overall stage (*P* = .035) when the patients were stratified by CNN.

In our study, all NPC patients with positive lymph node metastasis. We measured the maximum diameter of lymph node. Among them, 17.6% (107/607) individuals had a maximum diameter in excess 27 mm. Of the 183 CNN patients, there were 62.6% (67/183) patients who reached the maximum diameter of the lymph node exceeding 27 mm. In the 424 non‐CNN patients, only 40 (9.4%) patients exceeded 27 mm for the maximum diameter of lymph node. However, in the CNN group, 67 (62.6%) patients achieved or exceeded that size. Furthermore, CNN was significantly related to the maximum diameter of lymph node, with a correlation coefficient of .327 (*P* < .001).

**Figure 4:**
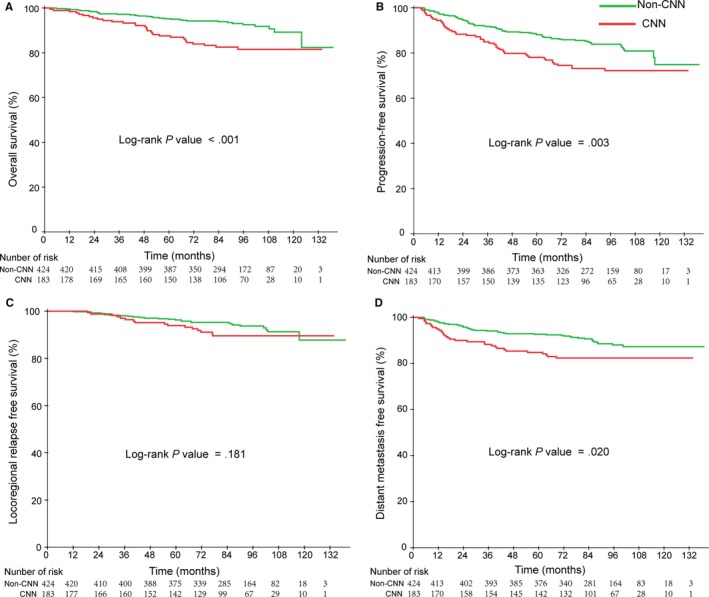
Kaplan‐Meier curves of overall (A), Progression‐free (B), Locoregional relapse‐free (C), and Distant metastasis free (D) survival outcomes for the 607 NPC patients stratified by CNN status

In the CNN group, 16.9% (31/183) of patients died, while 16/183 (8.7%) and 31/183 (16.9%) developed local regional recurrence and distant metastases, respectively. In contrast, in the non‐CNN group, only 6.4% (27/424) and 10.8% (46/424) developed local regional recurrence and distant metastases, respectively, while 7.5% (32/424) patients died. Similarly, patients in the CNN group had shorter OS, PFS, and DMFS compared with those in the non‐CNN group. The 5‐year OS, PFS, and DMFS for the non‐CNN group and CNN group were 95.0% vs 86.9%, 88.0% vs 77.8%, and 92.5% vs 84.2%, with corresponding *P*‐values of .000, .003, and .020, respectively. However, there was no significant difference in the 5‐year LRFS between the CNN and non‐CNN group (*P* = .181). Kaplan‐Meier survival curves for the four groups are shown in Figure 4.

When entered into multivariate analysis, gender, T stage, N stage, EBV DNA level, and CNN were significant independent negative prognostic factors for survival. Multivariate analyses also demonstrated that CNN was an independent prognostic factor for OS (HR = 1.927, 95% CI: 1.129‐3.290, *P* = .016), PFS (HR = 1.492, 95% CI: 1.100‐2.328, *P* = .014), and DMFS (HR = 1.661, 95% CI: 1.044‐2.644, *P* = .016). However, LRFS was not a significant prognostic factor for OS.

### 3.4 Association of EBV DNA level and CNN

Using Spearman correlation analysis, we found that the EBV DNA level correlated significantly with CNN, with a correlation coefficient of .324 (*P* < .001) **(**Table 2 **)**. We also found that the correlation analysis indicated that N classification correlated significantly with CNN, with a correlation coefficient of .203 (*P* < .001). The relationship between CNN and the maximum diameter of lymph node is closed, with a correlation coefficient of .327 (*P* < .001).

**Table 2:** The relationship between CNN and EBV DNA level

	EBV DNA ≤ 4000	EBV DNA > 4000	Total	*P*
Non‐CNN	313	111	424	
CNN	73	110	183	
Total	386	221	607	<.001

Abbreviation: CNN, cervical node necrosis.

### 3.5 Combination of EBV DNA level and CNN status improved prognostic stratification

**Figure 5:**
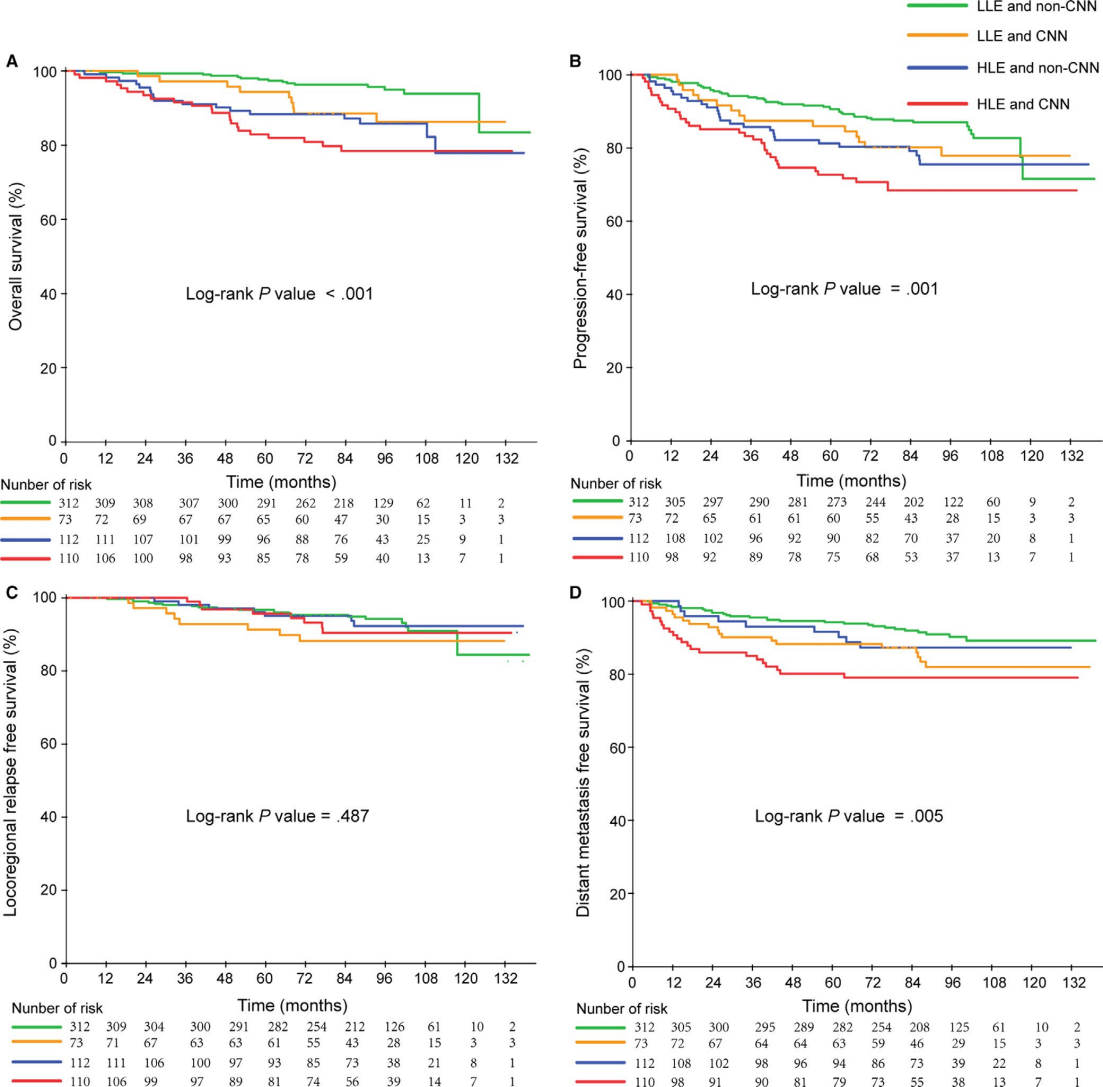
Kaplan‐Meier curves of overall (A), Progression‐free (B), Locoregional relapse‐free (C) and Distant metastasis‐free (D) survival outcomes for the 607 NPC patients stratified by EBV DNA level and CNN. *LLE and non‐CNN, low‐level EBV DNA and non‐CNN; LLE and CNN, low‐level EBV DNA and CNN; HLE and non‐CNN, high‐level EBV DNA and non‐CNN; HLE and CNN, high‐level EBV DNA and CNN

According to the aforementioned analysis, both EBV DNA level and CNN were independent prognostic factors for OS, PFS, and DMFS (Table 3). Therefore, we stratified the entire population into four groups using the two prognostic factors of pretreatment EBV DNA level and CNN: low‐level EBV DNA and non‐CNN group (LLE and non‐CNN), low‐level EBV DNA and CNN group (LLE and non‐CNN), high‐level EBV DNA and non‐CNN group (HLE and CNN), and high‐level EBV DNA and CNN group (HLE and CNN). Among those patients, the proportion of LLE and non‐CNN, LLE and CNN, HLE and non‐CNN, and HLE and CNN were 51.4% (313 of 607), 12.0% (73 of 607), 18.5% (111 of 607), 18.1% (110 of 607), respectively. In these four groups, the 5‐year OS was 97.4%, 93.3%, 88.1%, and 81.9% (*P*_trend_ < .001); 5‐year PFS was 90.5%, 85.0%, 80.7%, 72.1% (*P*_trend_ = .001); and the 5‐year DMFS was 94.1%, 90.1%, 87.9%, and 79.3% (*P* = .005), respectively. Kaplan‐Meier survival curves for the four groups are shown in Figure 5.

**Table 3:** Prognostic value of EBV DNA and CNN for OS, PFS, LRFS, and DMFS in the 607 NPC patients

Endpoint	Variable	HR (95% CI)*	*P* **
OS	**Gender**	**3.009 (1.289, 7.024)**	**.011**
	Age	1.154 (0.691, 1.926)	.585
	WHO pathology	0.917 (0.283, 2.972)	.886
	Smoking	1.200 (0.902, 1.596)	.210
	NPC family history	1.089 (0.533, 2.225)	.814
	T category	1.314 (0.670, 2.577)	.426
	**N category**	**2.467 (1.176, 5.179)**	**.017**
	**EBV DNA**	**2.157 (1.254, 3.710)**	**.005**
	**CNN(yes vs no)**	**1.927 (1.129, 3.289)**	**.016**
PFS	Gender	1.357 (0.851, 2.163)	.200
	Age	1.096 (0.769, 1.561)	.612
	WHO pathology	1.220 (0.495, 3.004)	.666
	Smoking	1.136 (0.922, 1.398)	.231
	NPC family history	0.843 (0.498, 1.427)	.525
	T category	1.174 (0.753, 1.828)	.479
	**N category**	**1.787 (1.005, 3.176)**	**.048**
	**EBV DNA**	**1.620 (1.120, 2.345)**	**.010**
	**CNN(yes vs no)**	**1.492 (1.100, 2.328)**	**.014**
LRFS	Gender	0.865 (0.400, 1.871)	.713
	Age	1.476 (0.800, 2.724)	.213
	WHO pathology	0.953 (0.227, 4.001)	.948
	Smoking	1.315 (0.913, 1.893)	.141
	NPC family history	0.933 (0.392, 2.219)	.875
	T category	0.796 (0.396, 1.601)	.522
	N category	0.643 (0.152, 2.717)	.548
	EBV DNA	0.878 (0.456, 1.691)	.697
	CNN(yes vs no)	1.671 (0.874, 3.193)	.120
DMFS	Gender	1.543 (0.848, 2.808)	.156
	Age	0.872 (0.562, 1.354)	.542
	WHO pathology	0.914 (0.331, 2.524)	.862
	Smoking	1.144 (0.885, 1.478)	.304
	NPC family history	0.947 (0.500, 1.794)	.868
	T category	1.712 (0.931, 3.147)	.084
	**N category**	**2.724 (1.438, 5.159)**	**.002**
	**EBV DNA**	**1.857 (1.169, 2.948)**	**.009**
	**CNN(yes vs no)**	**1.661 (1.044, 2.644)**	**.032**

Abbreviations: CI, confidence interval; DMFS, distant metastasis‐free survival; HR, hazard ratio; LRFS, locoregional relapse‐free survival; NPC, nasopharyngeal carcinoma; OS, overall survival; PFS, progression‐free survival.

Bold indicates *P* < .05

* The Cox proportional hazards model multivariate analysis includes the following variable: gender (male or female), age (≤44 vs >44 y), WHO pathology (type II vs type III), Smoking or nonsmoking, NPC family history, T category (T1‐2 vs T3‐4), N category (N1 vs N2‐3), EBV DNA levels (≤4000 vs >4000 copies), CNN status (non‐CNN vs CNN).

** *P* values were calculated by using the log‐rank test.

From the Figure 5, the high‐level EBV DNA and CNN group had the poorest survival outcomes, while the low‐level EBV DNA and non‐CNN group had the best survival outcomes. Compared with the low‐level EBV DNA and non‐CNN group, the high‐level EBV DNA and CNN group had poor OS, PFS, and DMFS.

Subgroup analyses of combined EBV DNA level and CNN according to the clinical stage are presented in Table 4. The survival outcomes for these with clinical stage IV disease and high‐level EBV DNA and CNN were substantially poor than those patients in clinical stage II or III, with or without CNN and high‐level EBV DNA. However, the combination of EBV DNA and CNN did not substantially affect the local‐regional recurrence in all clinical stage III patients.

**Table 4:** Subgroup analysis of combined EBV DNA and CNN groups in different clinical stages

Endpoint	Subgroup	LLE and non‐CNN*	LLE and CNN*	HLE and non‐CNN*	HLE and CNN*	*P*_trend_
OS	Clinical stage II+III	97.9	96.1	85.3	87.7	<.001
	IV	93.9	74.3	89.1	71.5	.008
PFS	Clinical stage II+III	92.0	87.5	77.3	78.5	.010
	IV	83.5	65.5	86.2	55.4	.003
LRFS	Clinical stage II+III	96.3	91.2	94.8	95.1	.605
	IV	96.8	85.3	90.9	89.9	.839
DMFS	Clinical stage II+III	95.6	91.9	84.8	87.5	.006
	IV	87.2	69.1	89.9	62.7	.003

Abbreviations: HLE and CNN, high‐level EBV DNA and CNN group; HLE and non‐CNN, high‐level EBV DNA and non‐CNN group; LLE and CNN, low‐level EBV DNA and CNN group; LLE and non‐CNN, low‐level EBV DNA and non‐CNN group.

* Numbers in parentheses are percentages.

In multivariate analysis, the combined classification was demonstrated to be an independent prognostic factor for OS (*P* < .001), PFS (*P* = .001), and DMFS (*P* = .018). In the high‐level EBV DNA and CNN group, NPC patients had a high rist for OS (HR, 4526; 95% CI: 2.319, 8.835), for PFS (HR, 2.473; 95% CI: 1.572,3.890), and for DMFS (HR, 2.473; 95% CI: 1.572, 4.286).

## 4 DISCUSSION

To the best of our knowledge, our study is the first retrospective study that combined pretreatment plasma EBV DNA level and CNN status to assess the prognosis of NPC patients. We observed a significant association between plasma EBV DNA level and CNN status in the IMRT era, which we believe has important clinical relevance. In a previous study, Lan et al demonstrated that cervical nodal necrosis was an independent negative prognostic factor in NPC patients.^16^ Our study indicated that CNN status and also combination of pretreatment plasma EBV DNA level and CNN status are strongly associated with survival outcome in NPC patients.

The detection of tumor‐associated DNA, such as plasma EBV DNA in NPC, has created new possibilities for early detection of tumor. More than 95% of patients with NPC can be tested for this plasma marker by quantitative polymerase chain reaction, a rapid and sensitive technique.^9^, ^23^ According to previous studies, pretreatment EBV DNA is one of the most significant prognostic factor of NPC, and is clinically employed for the diagnosis, risk classification, dynamic monitoring, and prognosis of NPC.^5^, ^6^, ^7^, ^8^, ^9^, ^19^ As other group concluded, one of the origins of EBV DNA that is detectable in plasma, is derived from tumor cell death.^10^, ^11^ In addition, many clinicians accept that pretreatment plasma EBV DNA reflects the NPC gross tumor burden, and the level of plasma EBV DNA is strongly correlated with the TNM classification and overall stage.^18^, ^24^, ^25^, ^26^, ^27^, ^28^, ^29^ Many studies have demonstrated that pretreatment EBV DNA with a cutoff value at 4000 copies/mL has a better prognostic value.^19^ In our study, we also employed this optimal cut‐off, and found that high‐level EBV DNA predicted high risk and worse survival outcome, while pretreatment plasma EBV DNA > 4000 copies/mL had a worse 5‐year OS, PFS, and DMFS. This result is similar to a previous study.^11^, ^30^

Recent evidence indicates CNN is a negative prognostic factor in NPC.^16^ Don and colleagues previously reported that central necrosis is correlated with increasing nodal size,^31^ and is a signal for tumor metastases in head and neck squamous cell carcinoma.^32^, ^33^, ^34^, ^35^, ^36^, ^37^ In our study, we also found that patients in the CNN group had a poor survival outcome, compared with the non‐CNN group. As mentioned above, tumor necrosis may indicate tumor hypoxia and the most important source of plasma EBV DNA is tumor cell death. Consequently, we postulated that there may be an association between EBV DNA level and CNN status. After we performed correlation analysis, we found that EBV DNA level correlated significantly with CNN, with a correlation coefficient of .324. Generally, the larger tumor volume, the more likely it is that there would be high‐level EBV DNA and necrosis.

Although there are many prognostic factors in NPC, distant metastasis is the main reason for treatment failure. Consequently, we performed a large retrospective study to determine the combined prognostic value of EBV DNA level and CNN status in an attempt to elucidate more effective treatment regimens. Based on this work, we propose a prognostic model to assess new stratification of individual NPC patients receiving IMRT treatment into four risk groups. The low‐level EBV DNA and non‐CNN group of patients had better survival outcome than the low‐level EBV DNA and CNN group, the high‐level EBV DNA and non‐CNN group, and especially the high‐level EBV DNA and CNN group. Patients with a high‐level EBV DNA and CNN status had a significantly higher 4.5‐fold risk of death (HR, 4.526; 95% CI: 2.319‐8.835), significantly higher PFS (HR, 2.473; 95% CI: 1.572‐3.890), and significantly high DMFS (HR, 2.473; 95% CI: 1.572‐4.286) than those patients in the low‐level EBV DNA and non‐CNN group. The combined stratification was an independent and negative factor for OS, PFS, and DMFS. It appears that the combination of factors improved prognostic ability.

NPC is both a radiosensitive and chemosensitive carcinoma and clinical outcome tends to be encouraging, compared with other cancer where radio‐ and chemoresistance are problematic.^38^ In our study, we demonstrated that in patients with high‐level EBV DNA and CNN status, who were a high‐risk group, had worse survival than other groups. While the exact mechanism associated with poor survival in the high‐risk group is not clear, it could appear that an increase in the intensity of therapy can be made according to our classification.

However, there are some drawbacks to our study. The first limitation is the assessment of CNN was reached by two or three radiologists who specialized in head and neck cancer and they reviewed all MRI scans separately. Furthermore, they used this diagnostic modality, rather than cervical nodal biopsy, which is the most informative examination but invasive. The second limitation is that all NPC patients originated from one single institution. Therefore, more population‐based studies are required to confirm our conclusions.

## 5 CONCLUSION

The status of cervical lymph nodes and pretreatment plasma EBV DNA level are negative prognostic factors and both were closely correlated in NPC patients. During the IMRT era, a combination of pretreatment EBV DNA level and CNN status can improve risk stratification and prognostic value allowing available and noninvasive examinations to be performed.

## AUTHOR CONTRIBUTIONS

Study concepts: Hao‐Yuan Mo, Wen‐Hui Chen; Study design: Yu‐Yun Du, Dong‐Hua Luo, Xue‐Song Sun; Data acquisition: Yu‐Yun Du, Dong‐Hua Luo, Xue‐Song Sun, Lin‐Quan Tang, Hai‐Qiang Mai, Qiu‐Yan Chen, Jing‐Hua Zhong, Dong‐Mei Mai, Wan‐Ru Zhang; Quality control of data and algorithms: Yu‐Yun Du, Jing‐Hua Zhong; Data analysis and interpretation: Yu‐Yun Du, Xue‐Song Sun, Dong‐Hua Luo; Statistical analysis: Yu‐Yun Du, Xue‐Song Sun, Dong‐Hua Luo; Manuscript preparation: Yu‐Yun Du, Dong‐Hua Luo, Xue‐Song Sun; Manuscript editing: Yu‐Yun Du, Dong‐Hua Luo, Xue‐Song Sun; Manuscript review: Hao‐Yuan Mo, Lin‐Quan Tang, Hai‐Qiang Mai, and Wen‐Hui Chen.

## Data Availability

The datasets used and/or analyzed during the current study are available from the corresponding author on reasonable request.

## References

[1] Ho FC , Tham IW , Earnest A , Lee KM , Lu JJ . Patterns of regional lymph node metastasis of nasopharyngeal carcinoma: a meta‐analysis of clinical evidence. BMC Cancer. 2012;12:98. 22433671 10.1186/1471-2407-12-98 PMC3353248

[2] Loong HH , Ma BB , Chan AT . Update on the management and therapeutic monitoring of advanced nasopharyngeal cancer. Hematol Oncol Clin North Am. 2008;22:1267‐1278. 19010273 10.1016/j.hoc.2008.08.012

[3] Lee AW , Fee WE Jr , Ng WT , Chan LK . Nasopharyngeal carcinoma: salvage of local recurrence. Oral Oncol. 2012;48:768‐774. 22425246 10.1016/j.oraloncology.2012.02.017

[4] Kam M , Teo P , Chau R , et al. Treatment of nasopharyngeal carcinoma with intensity‐modulated radiotherapy: the Hong Kong experience. Int J Radiat Oncol Biol Phys. 2004;60:1440‐1450. 15590175 10.1016/j.ijrobp.2004.05.022

[5] Chan A , Dennis Lo YM , Zee B , et al. Plasma Epstein‐Barr virus DNA and residual disease after radiotherapy for undifferentiated nasopharyngeal carcinoma. J Natl Cancer Inst. 2002;94:1614‐1619. 12419787 10.1093/jnci/94.21.1614

[6] Leung S‐F , Chan A , Zee B , et al. Pretherapy quantitative measurement of circulating Epstein‐Barr virus DNA is predictive of posttherapy distant failure in patients with early‐stage nasopharyngeal carcinoma of undifferentiated type. Cancer. 2003;98:288‐291. 12872347 10.1002/cncr.11496

[7] Leung S , Zee B , Ma BB , et al. Plasma Epstein‐Barr viral deoxyribonucleic acid quantitation complements tumor‐node‐metastasis staging prognostication in nasopharyngeal carcinoma. J Clin Oncol. 2006;24:5414‐5418. 17135642 10.1200/JCO.2006.07.7982

[8] Lin J , Wang WY , Chen KY , et al. Quantification of plasma Epstein‐Barr virus DNA in patients with advanced nasopharyngeal carcinoma. N Engl J Med. 2004;350:2461‐2470. 15190138 10.1056/NEJMoa032260

[9] Lo YM , Chan LY , Lo KW , et al. Quantitative analysis of cell‐free Epstein‐Barr virus DNA in plasma of patients with nasopharyngeal carcinoma. Cancer Res. 1999;59:1188‐1191. 10096545

[10] Chan KC , Zhang J , Chan AT , et al. Molecular characterization of circulating EBV DNA in the plasma of nasopharyngeal carcinoma and lymphoma patients. Can Res. 2003;63:2028‐2032. 12727814

[11] Lo YM , Leung SF , Chan LY , et al. Kinetics of plasma Epstein‐Barr virus DNA during radiation therapy for nasopharyngeal carcinoma. Cancer Res. 2000;60:2351‐2355. 10811107

[12] Chua D , Sham J , Kwong D , et al. Volumetric analysis of tumor extent in nasopharyngeal carcinoma and correlation with treatment outcome. Int J Radiat Oncol Biol Phys. 1997;39:711‐719. 9336154 10.1016/s0360-3016(97)00374-x

[13] Gatenby RA , Kessler HB , Rosenblum JS , et al. Oxygen distribution in squamous cell carcinoma metastases and its relationship to outcome of radiation therapy. Int J Radiat Oncol Biol Phys. 1988;14:831‐838. 3360652 10.1016/0360-3016(88)90002-8

[14] Groebe K , Mueller‐Klieser W . On the relation between size of necrosis and diameter of tumor spheroids. Int J Radiat Oncol Biol Phys. 1996;34:395‐401. 8567341 10.1016/0360-3016(95)02065-9

[15] Yaes RJ . Tumor heterogeneity, tumor size, and radioresistance. Int J Radiat Oncol Biol Phys. 1989;17:993‐1005. 2808062 10.1016/0360-3016(89)90147-8

[16] Lan M , Huang Y , Chen C‐Y , et al. Prognostic value of cervical nodal necrosis in nasopharyngeal carcinoma: analysis of 1800 patients with positive cervical nodal metastasis at MR imaging. Radiology. 2015;276:619. 10.1148/radiol.15154020 26203717

[17] Edge S , Compton C . The American Joint Committee on Cancer: the 7th edition of the AJCC cancer staging manual and the future of TNM. Ann Surg Oncol. 2010;17:1471‐1474. 20180029 10.1245/s10434-010-0985-4

[18] Shao J‐Y , Li Y‐H , Gao H‐Y , et al. Comparison of plasma Epstein‐Barr virus (EBV) DNA levels and serum EBV immunoglobulin A/virus capsid antigen antibody titers in patients with nasopharyngeal carcinoma. Cancer. 2004;100:1162‐1170. 15022282 10.1002/cncr.20099

[19] Tang LQ , Chen QY , Fan W , et al. Prospective study of tailoring whole‐body dual‐modality [18F]fluorodeoxyglucose positron emission tomography/computed tomography with plasma Epstein‐Barr virus DNA for detecting distant metastasis in endemic nasopharyngeal carcinoma at initial staging. J Clin Oncol. 2013;31:2861‐2869. 23857969 10.1200/JCO.2012.46.0816

[20] Liu L‐T , Tang L‐Q , Chen Q‐Y , et al. The prognostic value of plasma Epstein‐Barr viral DNA and tumor response to neoadjuvant chemotherapy in advanced‐stage nasopharyngeal carcinoma. Int J Radiat Oncol Biol Phys. 2015;93:862‐869. 26530755 10.1016/j.ijrobp.2015.08.003

[21] King AD , Tse G , Ahuja AT , et al. Necrosis in metastatic neck nodes: diagnostic accuracy of CT, MR imaging, and US. Radiology. 2004;230:720‐726. 14990838 10.1148/radiol.2303030157

[22] Chung MS , Cheng KL , Choi YJ , et al. Interobserver reproducibility of cervical lymph node measurements at CT in patients with head and neck squamous cell carcinoma. Clin Radiol. 2016;71:1226‐1232. 27569854 10.1016/j.crad.2016.07.014

[23] Loghavi S . Quantitative PCR for plasma Epstein‐Barr virus loads in cancer diagnostics. Methods Mol Biol. 2016;1392:51‐61. 26843046 10.1007/978-1-4939-3360-0_6

[24] Han B , Xu XY , Zhang CZ , et al. Systematic review on Epstein‐Barr virus (EBV) DNA in diagnosis of nasopharyngeal carcinoma in Asian populations. Asian Pac J Cancer Prev. 2012;13:2577‐2581. 22938423 10.7314/apjcp.2012.13.6.2577

[25] Baizig NM , Morand P , Seigneurin JM , et al. Complementary determination of Epstein‐Barr virus DNA load and serum markers for nasopharyngeal carcinoma screening and early detection in individuals at risk in Tunisia. Eur Arch Otorhinolaryngol. 2012;269:1005‐1011. 21805179 10.1007/s00405-011-1717-5

[26] Chai SJ , Pua KC , Saleh A , et al. Clinical significance of plasma Epstein‐Barr Virus DNA loads in a large cohort of Malaysian patients with nasopharyngeal carcinoma. J Clin Virol. 2012;55:34‐39. 22739102 10.1016/j.jcv.2012.05.017

[27] Lo Y , Leung SF , Chan L , et al. Plasma cell‐free Epstein‐Barr virus DNA quantitation in patients with nasopharyngeal carcinoma. correlation with clinical staging. Ann NY Acad Sci. 2000;906:99‐101. 10818603 10.1111/j.1749-6632.2000.tb06597.x

[28] Chan K , Hung E , Woo J , et al. Early detection of nasopharyngeal carcinoma by plasma Epstein‐Barr virus DNA analysis in a surveillance program. Cancer. 2013;119:1838‐1844. 23436393 10.1002/cncr.28001

[29] Sun P , Chen C , Cheng Y‐K , et al. Serologic biomarkers of Epstein–Barr virus correlate with TNM classification according to the seventh edition of the UICC/AJCC staging system for nasopharyngeal carcinoma. Eur Arch Otorhinolaryngol. 2014;271(9):2545‐2554. 24213277 10.1007/s00405-013-2805-5

[30] Chen W‐H , Tang L‐Q , Guo S‐S , et al. Prognostic value of plasma Epstein‐Barr virus DNA for local and regionally advanced nasopharyngeal carcinoma treated with cisplatin‐based concurrent chemoradiotherapy in intensity‐modulated radiotherapy era. Medicine. 2016;95:e2642. 26844482 10.1097/MD.0000000000002642 PMC4748899

[31] Don DM , Anzai Y , Lufkin RB , Fu YS , Calcaterra TC . Evaluation of cervical lymph node metastases in squamous cell carcinoma of the head and neck. Laryngoscope. 1995;105:669‐674. 7603268 10.1288/00005537-199507000-00001

[32] Randall DR , Lysack JT , Hudon ME , et al. Diagnostic utility of central node necrosis in predicting extracapsular spread among oral cavity squamous cell carcinoma. Head Neck. 2015;37:92‐96. 24327459 10.1002/hed.23562

[33] Johnson JT , Myers EN , Bedetti CD , Barnes EL , Schramm VL Jr , Thearle PB . Cervical lymph node metastases. incidence and implications of extracapsular carcinoma. Arch Otolaryngol. 1985;111:534‐537. 4026664 10.1001/archotol.1985.00800100082012

[34] Snyderman NL , Johnson JT , Schramm VL , Myers EN , Bedetti CD , Thearle P . Extracapsular spread of carcinoma in cervical lymph nodes. impact upon survival in patients with carcinoma of the supraglottic larynx. Cancer. 1985;56:1597‐1599. 4027895 10.1002/1097-0142(19851001)56:7<1597::aid-cncr2820560722>3.0.co;2-5

[35] Brasilino de Carvalho M . Quantitative analysis of the extent of extracapsular invasion and its prognostic significance: a prospective study of 170 cases of carcinoma of the larynx and hypopharynx. Head Neck. 1998;20:16‐21. 9464947 10.1002/(sici)1097-0347(199801)20:1<16::aid-hed3>3.0.co;2-6

[36] Puri SK , Fan CY , Hanna E . Significance of extracapsular lymph node metastases in patients with head and neck squamous cell carcinoma. Curr Opin Otolaryngol Head Neck Surg. 2003;11:119‐123. 14515090 10.1097/00020840-200304000-00010

[37] Jose J , Coatesworth AP , Johnston C , MacLennan K . Cervical node metastases in squamous cell carcinoma of the upper aerodigestive tract: the significance of extracapsular spread and soft tissue deposits. Head Neck. 2003;25:451‐456. 12784236 10.1002/hed.10214

[38] Harrison L , Blackwell K . Hypoxia and anemia: factors in decreased sensitivity to radiation therapy and chemotherapy? Oncologist. 2004;9(Suppl 5):31‐40. 15591420 10.1634/theoncologist.9-90005-31

